# Factors Influencing the Expansion of Arch-Shaped Electromagnetic Railguns with Pre-Stressed Composite Barrels

**DOI:** 10.3390/ma16165535

**Published:** 2023-08-09

**Authors:** Junsheng Wang, Jun Xiao, Dajun Huan, Lei Yan

**Affiliations:** R&D Center for Composites Industry Automation, College of Material Science and Technology, Nanjing University of Aeronautics and Astronautics, Nanjing 210016, China; js_wong@nuaa.edu.cn (J.W.); huandj@nuaa.edu.cn (D.H.); bx2006315@nuaa.edu.cn (L.Y.)

**Keywords:** rail expansion, finite element method, pre-stressed composite barrel

## Abstract

Rail expansion significantly impacts the launch precision of a railgun system. Higher precision can be achieved when the extent of expansion is low. This paper investigates three main factors that influence the extent of rail expansion using the finite element method, including pre-stress, electromagnetic load, and stiffness of the insulators. The mean squared error between experiment results and simulating results is less than 0.06, validating the finite element model. The simulated results reveal that the extent of rail expansion increases with a decrease in pre-stress and an increase in electromagnetic pressure and the stiffness of the insulator is the most significant influencing factor, as the use of a stiff insulator not only results in a small extent of rail expansion but also delays the separation between the rails and insulators. The mechanism of how pre-stress influences the railgun system has been proposed. It has been expressed that the pre-stress maintains the integrity of the railgun system by hindering the process of a decrease in the contact surface area between rails and insulators during launch. The study provides a platform to improve the design of the railgun system.

## 1. Introduction

**Figure 1:**
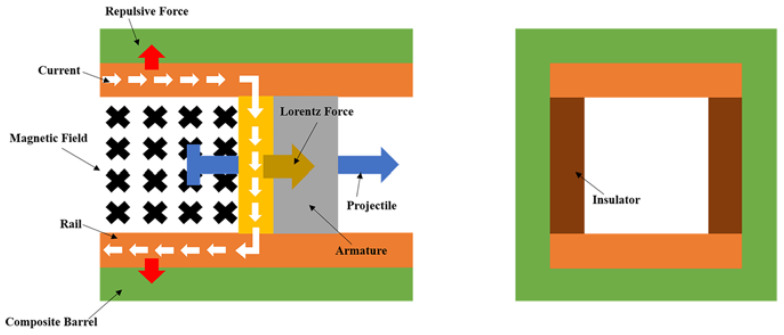
Schematic diagram of an electromagnetic railgun.

Electromagnetic railguns (EM guns) have garnered increasing attention over the past few decades due to their ability to assist in the process of hypervelocity launch [1]. Figure 1 illustrates the typical components of a railgun, which includes two rails, an armature, two insulators, and a composite barrel. During the launch procedure, the current from the power source is fed into one rail, flows through the armature, and finally returns to the power source through another rail. As a result, a magnetic field is generated, and the armature experiences the Lorentz force, causing it to accelerate along the rail. The projectile is connected to the armature, and when the armature moves out of the muzzle, the launch process is completed. In the launch process, apart from the Lorentz force acting on the armature, a repulsive force is generated between the two rails, leading to rail expansion. The rail expansion can cause the armature to disconnect from the rails, resulting in poor launch performance. Previous reports have highlighted the significant influence of railgun stiffness on launch performance [2,3,4,5]. However, researchers have been more interested in material selection [6], cross-section [7], and the moment of inertia [8] of a single component. Surprisingly the overall structural stiffness, which plays a crucial role in system performance, has received little attention. Moreover, most studies on structural design and optimization have focused on the interaction between the armature and rails [6,7,8,9,10,11,12,13]. It has been reported that the contact relationship between the armature and rails plays a key role in launch performance [14,15,16,17] by affecting the velocity and precision of the system [18,19,20,21,22,23]. However, few studies have investigated the relationship between rails and insulators. The concept of pre-stressing has been employed to maintain the geometric configuration of assembled railgun components during launch [24]. By applying pre-stress, compression stress is generated, counteracting the electromagnetic forces acting on the system. Several methods have been researched to achieve pre-stress in railguns [25,26,27], including interference fitting [28], bolt pre-tightening [14], laminate containment prestress, and pre-stressed winding [29]. Among these methods, bolt pre-tightening and filament winding are the most commonly used for achieving pre-stress in railguns equipped with composite barrels. However, studies on pre-stressed barrels have primarily focused on the stress distribution of the barrel [29,30,31,32,33,34], and there have been few investigations into the mechanism of how pre-stress influences the railgun system.

The influence of pre-stressed composite barrels on railguns has been extensively studied. However, most of these studies have focused on the interactions between the armature and rails, neglecting the significant interaction between the insulators and rails by assuming that they are bonded perfectly [34]. This oversight can impact the integrity of the railguns and potentially lead to issues such as aluminum from the armature melting and depositing onto the insulator, causing rail corrosion [35]. Additionally, it is worth noting that previous studies on pre-stressed barrels have not presented the mechanisms through which pre-stress influences railguns.

To address the relationship between the rails and the insulators during loading process and understand the mechanism behind pre-stress’s influence on railgun systems, we conducted a study using a flat-shaped railgun equipped with a pre-stressed composite barrel, employing the finite element method (FEM). Our investigation focuses on rail expansion and its influencing factors. We also utilize a simple physical model to illustrate the effect of the interaction between insulators and rails on rail expansion. The paper is organized into several sections. In Section 2, we present the winding–loading physical model and describe its mechanical behavior. Furthermore, we establish a finite element model of a railgun equipped with a pre-stressed composite barrel. Section 3 delves into the effects of three factors: pre-stress, electromagnetic loading, and the modulus of insulators on rail expansion, as well as their impact on the system’s performance. In this section, we also elaborate on the mechanism through which pre-stress influences the railgun system. Finally, in Section 4, we provide concluding remarks based on our findings and insights from the study.

## 2. Materials and Methods

Two methods (the physical modeling method and the finite element method) have been proposed in this section to illustrate the mechanical behavior of the railgun system during winding and loading process.

### 2.1. Winding–Loading Model

**Figure 2:**
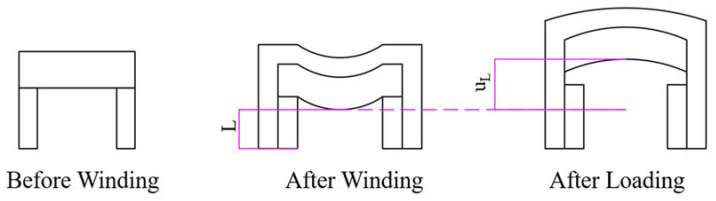
Description of rail expansion.

Rail expansion is defined as the percentage of displacement that occurs during loading relative to the initial dimension of the railgun’s caliber. It can be expressed as follows (illustrated in Figure 2):

(1) $$RE=\frac{u_{L}}{L}\times 100\%$$

where RE denotes rail expansion, L is the initial length of the caliber along the direction of electromagnetic load before loading, and $u_{L}$ is the displacement along the direction of electromagnetic load during loading. It is obvious that only the value of $u_{L}$ is unknown and needs to be determined, and it is affected by the pre-stress winding and loading procedure associated with a railgun equipped with a pre-stressed composite barrel. The mechanical procedure can be classified into two parts (the winding and the loading processes) to identify the factors that influence rail expansion after loading.

#### 2.1.1. Rail Displacement Achieve during the Filament Winding Process

**Figure 3:**
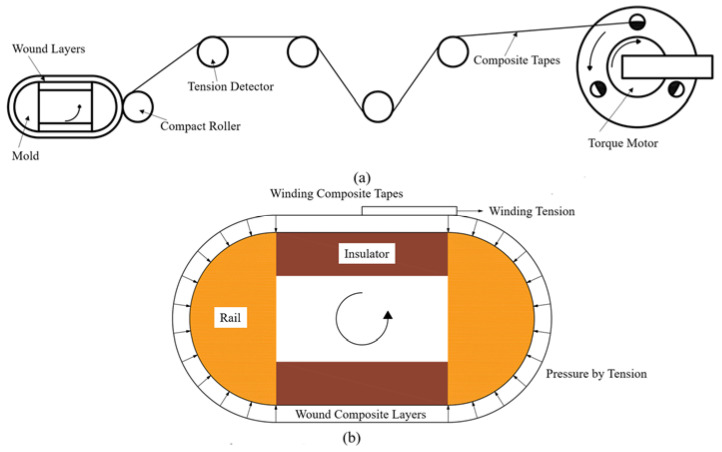
Pre-stressed winding process for the arch-shaped railgun. (**a**) Pre-stressed winding procedure. (**b**) Description of winding layer changing into external pressure.

**Figure 4:**
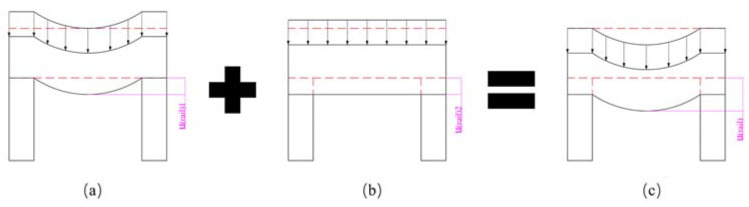
Sketch presenting the simplified deformed model used during the railgun winding procedure. (**a**) Insulator as the rigid body. (**b**) Rail as the rigid body. (**c**) Rail and insulator as the deformable bodies.

Pre-stressed winding, in which the pre-stress is applied on the filament while winding, is a common process to form pre-stressed composite barrels for railgun systems. This procedure is depicted in Figure 3a. The pre-stress on the tapes is converted into external pressure on the curved surfaces of the rails and insulators during this process, as depicted in Figure 3b. As mentioned earlier, external pressure is generated on the curved outer surface of the rail, and pressure is not generated at the inner straight area of the insulator in a railgun system. This follows winding law. Thus, the pre-stress on the composite tapes is converted into external pressure on the rail while the insulators remain unpressurized during winding. Therefore, the rails can be treated as beams (subject to bending load), while the insulators can be considered as compression rods (Figure 4). The rails are considered rectangular beams for simplification. The region enclosed by dotted lines in Figure 4 presents the original shapes of the rails and insulators, while the area enclosed by solid lines presents deformation.

The rails can be considered as supported beams subjected to uniform load if it is assumed that the insulators are rigid and the rails and insulators remain bonded (Figure 4a). Rail displacement approximately equals to beam deflection, and this can be expressed as follows (Equation (2)):

(2) $$u_{(rail)1}=\int \frac{\sigma_{(rail)y}(P)}{E_{rail}}dy\approx w_{prestress}=\int \frac{M(P)}{E_{rail}I_{rail}}dx$$

where $E_{rail}$ denotes the modulus of rail, $I_{rail}$ denotes the inertia of rail, M(P) denotes the moment by external pressure (P), $u_{(rail)1}$ denotes the equivalent displacement of rail associated with the winding procedure when the insulator is considered as a rigid body, $w_{prestress}$ denotes the equivalent deflection of a beam associated with the winding procedure when the insulator is considered the rigid body, $\sigma_{(rail)y}(P)$ denotes the equivalent stress applied along the *Y*-direction of the beam during the winding procedure.

The insulator can be considered a compression member if it is assumed that the rail is a rigid body (Figure 4b). The displacement of the insulator achieved during the winding procedure is expressed as follows:

(3) $$u_{(insulator)1}=\int \frac{\sigma_{(rail)y}}{E_{insulator}}dy\approx \int \frac{F(P)}{E_{insulator}A_{insulaor}}dy$$

According to the continuity hypothesis, rail displacement can be expressed as follows:

(4) $$u_{(rail)2}=u_{(insulator)1}(y=\frac{h_{insulator}}{2})$$

where $E_{insulator}$ denotes the modulus associated with the insulator, $A_{insulator}$ denotes the cross-sectional area of the insulator, F(P) denotes the axial force exerted by external pressure P, $u_{(insulator)1}$ denotes the equivalent *Y*-direction displacement of the insulator during the winding procedure, $u_{(rail)2}$ denotes the equivalent *Y*-direction displacement of the rail during the winding procedure when the rail is considered as a rigid body, and $h_{insulator}$ denotes the length of the insulator.

As rails and insulators are deformable bodies, rail displacement achieved during the winding procedure can be expressed as follows:

(5) $$u_{\left(rail\right)}=u_{(rail)1}+u_{(rail)2}$$

#### 2.1.2. Rail Displacement Achieve during the Loading Process

**Figure 5:**
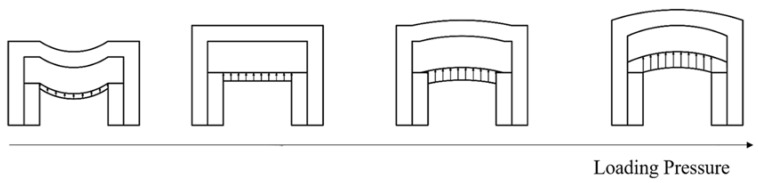
Sketch representing the simplified deformed model used to study railguns subjected to loading pressure.

The electromagnetic force is exclusively applied to the rails during the loading procedure, as depicted in Figure 1. The electromagnetic force, which is a type of body force, was considered surface pressure for simplicity. The entire railgun system can be considered an arch-shaped vessel under these conditions (Figure 5). It is challenging to analytically determine the precise displacement of the vessel [36]. However, the final displacement is primarily influenced by the modulus and the radius of the circular part of the arch-shaped cross-section. Therefore, the finite element method was used to arrive at the results reported in the following section. Let us delve into a qualitative discussion at this stage.

Figure 5 reveals that the insulator separates from the rail as the loading force increases. Typically, the equivalent axial force exerted on the insulator (Equation (3)) weakens during the loading process. This can be attributed to the fact that the loading pressure acts in the direction opposite to the direction along which pressure is generated under conditions of winding pre-stress. As a result, deformation of insulator realized during the winding process can be reverted in the absence of loading pressure on the insulator. However, it should be noted that the insulator’s deformation can be completely reversed when the loading pressure reaches a certain magnitude. The compressive state of the system transforms into a tensile state under these conditions. Thus, it can be inferred that the interaction between the rails and the insulator significantly affects system performance. Typically, there are two types of interactions:

1. Hard contact. The rails and insulators are in contact but not bonded to each other. In this type of interaction, the area of contact decreases as the loading force increases. This can be attributed to the fact that the insulator deformation realized during the winding process is reversed completely. This occurs as the equivalent beam bends under the influence of the loading force. Consequently, the entire system cannot be approximated to an arch-shaped vessel.
2. Bonded. The rails and insulators are bonded together using adhesives during the manufacturing process. In this scenario, the stress state of the insulation transitions from compressive to tensile as the deformation of the insulator recovers, and the loading force continues to increase. At this point, the strength of the bonded interface becomes the dominant factor. The interface breaks when it is unable to withstand the increasing force. The rails and insulators separate from each other under these conditions. After separation, similar observations were made for the previously mentioned case.

In conclusion, the process of rail expansion in a pre-stressed arch-shaped electromagnetic gun is influenced by the winding and loading processes. The mechanical properties, such as modulus, area, and inertia, play important roles in determining the final extent of expansion realized. Factors such as winding tension (pre-stress) and loading force significantly affect the process. It is important to emphasize the interaction between the rails and insulators, as it can result in barrel disintegration. Therefore, the factors that influence rail expansion were also studied.

### 2.2. Finite Element Model for the Arch-Shaped EM Gun with a Pre-Stressed Composite Barrel

ABAQUS (version 6.14.4), a commercial software, was used to establish a finite element model and examine the factors discussed in Section 1. The finite element method can be effectively used to simulate the winding of the arch-shaped cross-section. However, certain specialized techniques are required to improve the accuracy of the calculations.

#### 2.2.1. Modeling Technology

Some special modeling technologies were used in conjunction with the finite element model to simulate the complex pre-stress filament winding process accurately.

- Thermal parameter method. A critical challenge in modeling pre-stressed filament winding involves simulating the winding tension accurately. The thermal parameter method is proposed to address this issue. In the finite element model, a fictitious linear coefficient of thermal expansion, aligned along the fiber direction, is defined to reflect the material properties of the winding tapes. When a new layer is wound, the corresponding elements are activated and loaded with a fictitious temperature magnitude. This magnitude is defined as follows:

(6) $$\sigma_{\theta }=E_{\theta }\alpha \Delta Te$$

where α and ΔTe are the fictitious liner thermal expansion and magnitude of temperature variation coefficients, respectively, and $\sigma_{\theta }$ and $E_{\theta }$ are the stress and modulus of the winding layers along the fiber direction, respectively. Winding tension is applied as the layer shrinks due to temperature variations. Hence, this method is also known as the equivalent cooling method.

**Figure 6:**
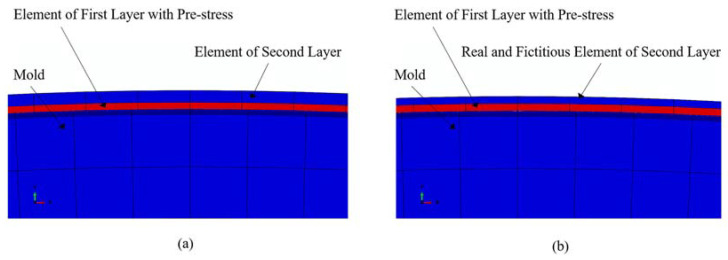
Modeling strategy. (**a**) Use of the element birth and death method. (**b**) Use of a combination of the element birth and death method and the real and fictitious element strategy.

- Element birth and death strategy. Another significant challenge involves simulating the layer-by-layer winding process accurately. The element birth and death strategy is used to address this issue. This strategy involves the deactivation of elements over certain steps and the activation of the elements over specific steps. The method allows for the modification of the layer-by-layer winding process. However, when the elements representing the outer layers are deactivated during the initial steps, they can still be influenced by the deformation of the inner layers under tension, as depicted in Figure 6a. This can introduce certain errors during simulation. The real–fictitious element analysis strategy is proposed to overcome this issue. This method involves assigning fictitious elements, which have the same node numbers as the specified real elements, with different material properties. Negligible deformation of the real elements, caused by the activation of neighboring elements, can be realized by ensuring that the stiffness of the fictitious elements is significantly smaller than that of the real elements. This can be attributed to the low stiffness of the fictitious elements, as depicted in Figure 6b. Hence, the element birth and death method, coupled with the real–fictitious element analysis strategy, is suitable for accurately simulating the pre-stress winding process.

#### 2.2.2. Model Establishment

**Figure 7:**
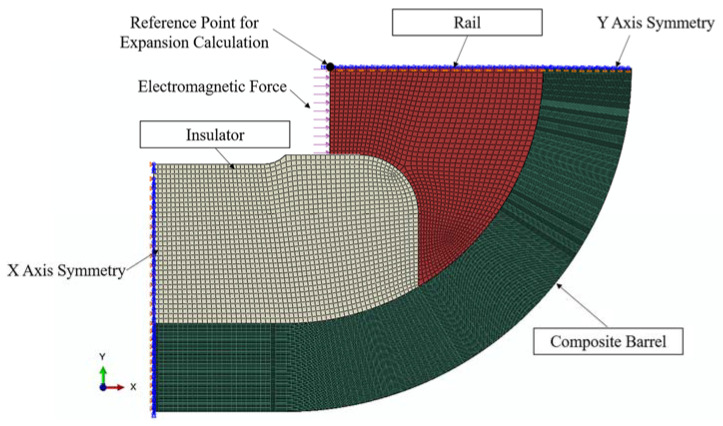
Finite element model for modified XMI railgun.

A 3D finite element model was developed to study the modified XMI electromagnetic railgun (Figure 7) to compare the results with previously reported experimental data [37]. A quarter model was used to take advantage of the symmetry of the railgun to simplify the computational process. The appropriate boundary conditions were applied, and the electromagnetic force (loading force) was approximated as a static external pressure on the bore (Figure 7). The C3D8R element type was used for all parts, and the dimension for the rail and insulator was approximately 1 mm^3^. The thickness of the winding layers was 0.2032 mm (0.008 inches), and this was comparable to the real thickness of the winding prepreg tapes. The thickness (along the *Z*-direction) of the model was recorded to be 25.4 mm (1 inch). A “soft spring connected to the ground” constraint was applied to both outer surfaces along the *Z*-direction (not shown in Figure 7) to prevent numerical singularities during simulation. This procedure ensures that the nodes at both outer surfaces are connected to the ground using low-stiffness springs. The purpose of these springs is to limit the displacement of the model along the *Z*-direction without significantly affecting the stress–strain state due to their low stiffness. Given that volume of each element of rail is 1 mm^3^, to ensure the contact surfaces of each part coordinate properly, the number of elements through the *Z*-direction is set to 25.

The rail material used in the study, as mentioned in [37], is off-the-shelf anode copper, while the insulator material is a standard off-the-shelf G-10 fiberglass block. The winding layers are composed of IM7/PEEK. The mechanical properties of these materials are presented in Table 1 and Table 2. A constant winding tension was applied (tension: 1135 N (250 lbs)) during the simulation, and the total thickness of the winding layers was 19.05 mm (0.75 inches). The prepreg width was 12.7 mm (0.5 inches) and consisted of 2 hoop layers and 1 axial layer. As the axial layer is subjected to negligible stress, the pre-stress can be expressed as follows:

(7) $$pre=\frac{T}{w\times t_{h}}=\frac{2}{3}\frac{T}{w\times t_{t}}$$

where T denotes the winding tension, pre denotes the pre-stress on the winding layers, w denotes the width of the winding layers, $t_{t}$ denotes the total thickness of the single layer, and $t_{h}$ denotes the thickness of the hoop layers in the single winding layer.

Pre-stress was also applied to the hoop layers using the composite layup function in ABAQUS. The displacement along the *X*-direction of the midpoint of the bore surface, which was subjected to electromagnetic forces, was used to calculate rail expansion (Figure 7). The dynamic launching process was assumed to be static to simplify the simulating procedure. Several steps were conducted to simulate the winding process using the strategies mention in Section 2.2.1. After the simulation of winding process finishes, the external pressure was applied to simulate the launch process. In conclusion, a 3D finite element model for the modified XMI railgun was developed to compare the obtained results with the previously reported experimental results and analyze the factors mentioned in Section 1.

## 3. Results and Discussion

In this section, the accuracy of the 3D finite element model is verified by comparing the results obtained using this model with the experimental results presented in Ref. [37]. The results obtained using the equivalent finite element model were analyzed. In this model, the thickness of a single winding layer is considered equivalent to the thickness of ten real winding layers. The results were found to be within the acceptable error range. Furthermore, the influencing factors, namely the modulus of the insulator, pre-stress, and loading force, were analyzed.

### 3.1. Verification of the 3D FE Model

**Figure 8:**
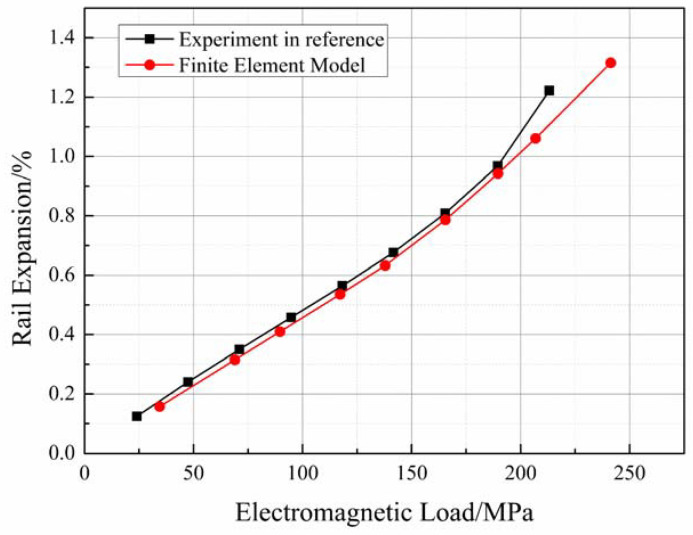
Comparison between the results obtained using the finite element model and the previously reported experimental results.

Rail expansion determined using the established 3D finite element model is calculated using Equation (8) as follows:

(8) $$RE=\frac{u_{E}-u_{p}}{L^{\prime }-u_{p}}\times 100\%$$

where RE is rail expansion realized, $L^{\prime }$ is the initial length of the bore before winding, and $u_{p}$ and $u_{E}$ are the final displacement after winding and loading, respectively. The results obtained using the finite element method and the experimental results are presented in Figure 8. The graphs were generated based on the results obtained using Equation (8) and the previously reported results [37].

Figure 8 demonstrates that the curve obtained using the 3D finite element model closely matches the curve obtained from the experimental results. The difference lays at the point corresponding to 213 MPa. The mean squared error (MSE) between the results obtained using the finite element model and the experimental results was less than 0.06, indicating a good agreement between the two.

A prominent and abrupt change in the profile of the experimental curve was observed. However, a similar change was not observed in the graph generated using the finite element model. This discrepancy can be attributed to the fact that the use of the off-the-shelf anode copper during the experiments resulted in a yield stress of 207 MPa. Therefore, the copper-based material yielded when the loading force reached 213 MPa. However, the finite element model does not consider the plasticity of the systems. Hence, a linear graph is obtained even when the force exceeds 213 MPa. It is important to emphasize that yielding behavior is not allowed during railgun loading. Therefore, the yielding behavior can be neglected while discussing the influencing factors associated with rail expansion. Consequently, it can be inferred that the 3D finite element model is well-suited for simulating railguns under the action of static loading forces and analyzing the factors that affect the process of rail expansion.

Figure 8 reveals another abrupt change in the experimentally obtained and finite element model-based curves at a loading of approximately 138 MPa. Unfortunately, there is insufficient experimental data to determine the cause of this change. However, the good fit of the results obtained using the finite element model with the available experimental data provides an opportunity to explore and explain this change. The details are discussed in the following subsection.

**Figure 9:**
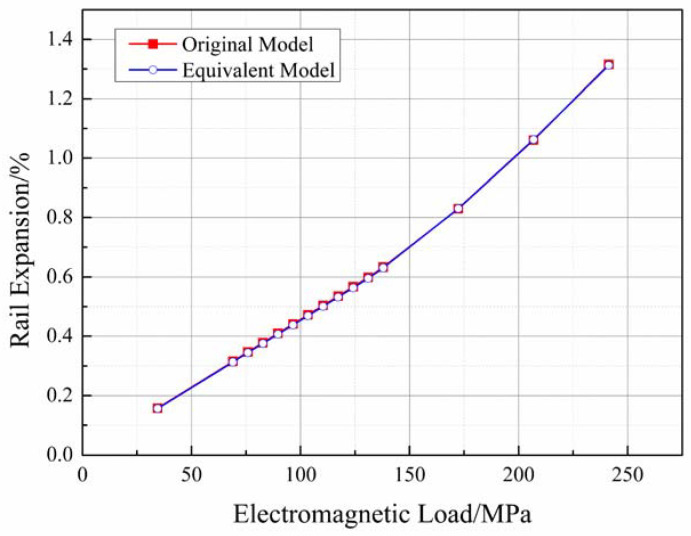
Comparison between the original and the equivalent models.

As the thicknesses of composite barrels of the model presented in Figure 8 are equal to the real thickness of a single layer, they cannot be used effectively for analysis in the presence of a large number of elements. The inefficiency can be attributed to the time-consuming nature of the process. This significantly increases the computational time. An equivalent model is introduced to improve computational efficiency. In the equivalent model, the thicknesses of the winding layers were adjusted to be 10 times higher than the thickness of a real single layer. This helped achieve the simultaneous simulation of ten winding layers. The results obtained from the original and equivalent models are compared and presented in Figure 9.

It can be observed that the two curves in Figure 9 are nearly identical, indicating a high level of agreement between the results obtained using the original and equivalent models. However, the computational time required for the equivalent model is only 1/50th of that required for the original model. This demonstrates that the equivalent model offers a significant time-saving advantage without compromising the accuracy of the results.

### 3.2. Mechanism of Pre-Stress Influences Railgun System

**Figure 10:**
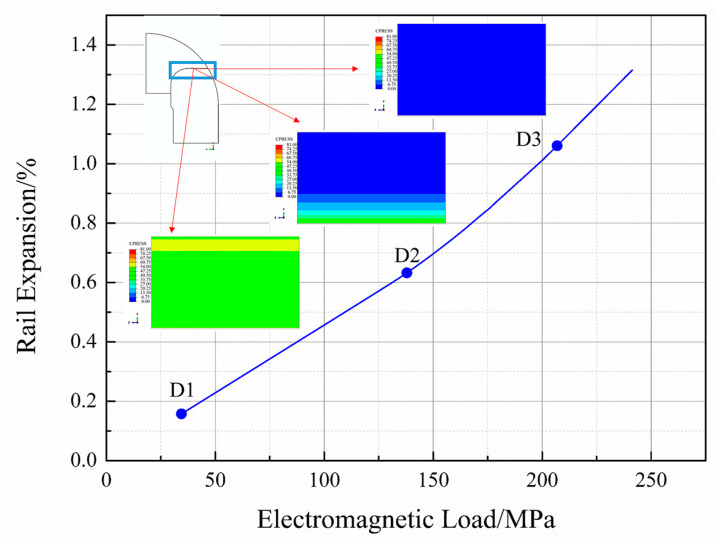
Electromagnetic load–rail expansion curve generated based on the finite element method.

It has been mentioned previously that the curves in Figure 9 present an abrupt change in profile when the loading force is approximately 138 MPa. Data were collected from the equivalent finite element model to rationalize this change, and the results are presented in Figure 10. The contour of the contact surface between the insulator and rail is presented in Figure 10 and the blue contour implies that the contact pressure drops to zero under these conditions.

Analysis of Figure 10 reveals that the curve appears linear when the loading force ranges between 34.5 and 117.2 MPa and 172.4 and 241.3 MPa. However, non-linear curves are generated when the loading force is within the range of 117.2–172.4 MPa. As mentioned in Section 2, the rails and insulators get deformed under conditions of pre-stress during the winding procedure. Electromagnetic forces solely act on the rail in the direction opposite to the direction of action of pre-stress during the loading procedure. This results in the reversal of deformation of the insulator caused during the winding procedure.

**Figure 11:**
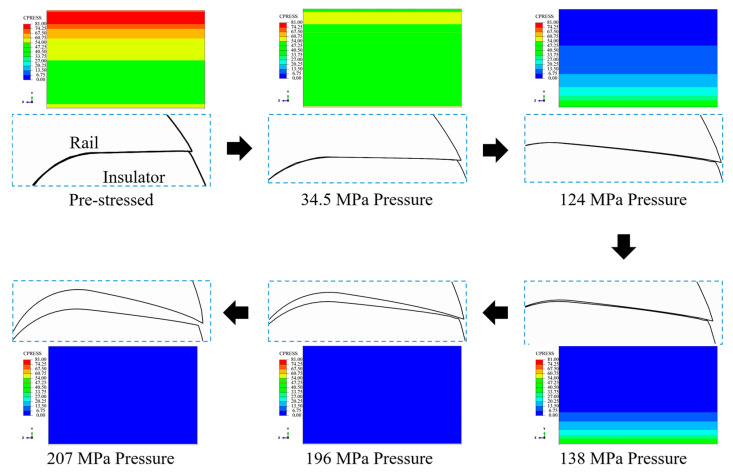
Graphs depicting the procedure of separation of the rail from the insulator under the condition of loading (contact pressure contour for the contact surface of the insulator).

The deformation of the insulator partially recovers when the loading force is relatively small. The entire system, including the rail, insulator, and winding layers, can be considered an integral unit, resulting in the generation of a linear graph. This corresponds to the linear region in the pressure range of 34.5–117.2 MPa (Part A). The deformation of the insulator continues to reverse as the loading force increases. Post-complete recovery, a further increase in the loading force results in the gradual separation between the insulator and the rail, and this can be attributed to rail bending. As a result, the contact area between the insulator and rail decreases (represented in blue), resulting in boundary nonlinearity. This is reflected by the nonlinear part of the curve (Part B). When the loading force continues to increase, the contact area between the rail and the insulator decreases until they separate completely (the contour of the contact surface is filled with blue). As a result, the condition of boundary nonlinearity disappears, and a linear graph is re-generated (Part C). However, it is important to note that the slope of linear Part A differs from that of linear Part C. This entire transition process is depicted in Figure 11. It can be seen from Figure 11 that the rail and insulator separate from each other with an increase in the electromagnetic pressure. The zero-contact pressure area on the contact surface (represented by the blue zone of the contour) gradually increases during this procedure. It indicates non-linear behavior during launching.

It is important to note that the curved surfaces corresponding to the rail and the insulator systems remain in contact during the loading procedure. This can be attributed to the curvature of the outer contour of the insulator, which applies external pressure on the curved part of the system and causes the insulator to squeeze the rail along the *Y*-direction during the winding process. This eventually results in deformation. System deformation cannot be fully recovered, and the contact pressure is maintained throughout the loading process as the electromagnetic force associated with the loading process acts along the *X* direction.

The effect of pre-stress on the barrel can be described as follows:

- Pre-stress is applied during the winding process through winding tension. This tension is transferred to external pressure on the outer surface of the curved part of the system, causing the deformation of the rails and the insulators.
- Contact pressure is generated at the interface between the rails and the insulators during winding procedure. The contact between the rails and the insulators is maintained during the loading process under conditions of deformation and the action of contact pressure. In other words, pre-stress is applied to the barrel to ensure that each component of the EM guns remains in contact during loading. This eventually helps maintain the stiffness and integrity of the system.

### 3.3. Factors Influencing the Extent of the Rail Expansion

As mentioned in Section 2 and the preceding discussion section, pre-stress, electromagnetic force, and the stiffness of each component are key factors that influence the process of rail expansion. In this subsection, we will discuss the effects of these factors associated with the finite element model on the modified XMI EM guns system. It is important to note that the rail is typically constructed using anode copper, and the use of a gun carriage often constrains the thickness of the winding layers. Therefore, it is impractical to delve into the two aforementioned factors. Hence, we will focus on three main factors (pre-stress, loading force, and the modulus of the insulators) that affect system performance.

#### 3.3.1. Effect of Pre-Stress

Pre-stress plays a crucial role in ensuring that the rail and the insulator remain in contact during the loading process. Therefore, it can be considered one of the most significant factors that affect the EM guns system. Previously presented examples revealed that the winding tension follows the constant tension method. The three commonly used winding tension control modes are constant tension, constant torque, and taper tension. The initial pre-stress for each layer of the three models can be expressed as follows:

(9) $$\sigma_{f}^{0(k)}=\sigma_{f}^{0(1)}(1-\beta \frac{R_{i}^{\left(k\right)}-R_{0}}{R_{i}^{\left(k\right)}})$$

where $\sigma_{f}^{0(1)}$ is the initial pre-stress along the fiber of the first layer, MPa, $\sigma_{f}^{0(k)}$ denotes the initial pre-stress along the fiber of the k-th layer, MPa, β is the taper coefficient (0≤β≤1), $R_{0}$ is the outer radius of the liner, mm, and $R_{i}^{\left(k\right)}$ denotes the inner radius of the kth layer, mm

**Figure 12:**
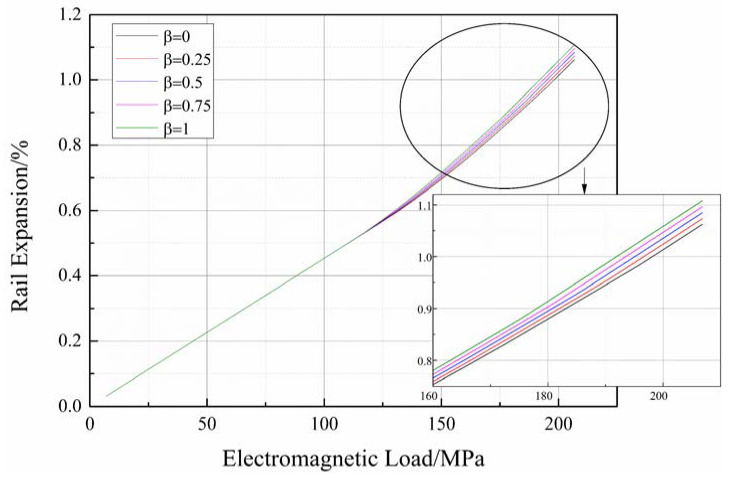
Comparison between different tension control modes.

For the modified XMI railgun system, $R_{0}$ is defined as the radius of the circular part of the rail. The radius was calculated to be 55.5625 mm. According to Equation (9), when β=0, the control mode transforms into the constant tension mode. When β=1, the mode transforms into the constant torque mode. These two modes were considered to arrive at the results reported herein. Three other modes (β=0.25, 0.5, and 0.75) have also been used to conduct the studies. The magnitude of the initial pre-stress along the fibers in the first layer was 290 MPa, and it remained constant under each mode. The results obtained for each control mode were compared, and the results are presented in Figure 12.

Figure 12 illustrates that the extent of rail expansion decreases with an increase in the taper coefficient β when the value of the electromagnetic load is over 125 MPa. Equation (9) expresses that the initial pre-stress of the k-th layer, $\sigma_{f}^{0(k)}$, decreases as β increases. According to the law of pre-stressed winding, a low pre-stress in the composite layer results in reduced external pressure on the curved surface of the winding mold. Analysis of the model discussed in Section 2 reveals that a low external pressure results in a decrease in the extent of deflection realized. This eventually results in an increase in the extent of rail expansion realized. The observations demonstrate that the winding tension can be increased to effectively reduce the extent of rail expansion realized and improve the firing precision of the railguns that are developed using pre-stressed winding composite wraps.

It is necessary to note that the two feature points indicating a partial and complete separation between rails and insulators, as mentioned in Section 3.2, differ across different modes. Figure 12 illustrates that the corresponding values of the electromagnetic load at both points are low for the larger taper coefficient. For instance, when β=1, the value of the electromagnetic load for the partially separated feature point is 107 MPa, whereas, for the completely separated point, it is 175 MPa. Conversely, when β=0, the corresponding values are 118 and 196 MPa, respectively. The reason behind this has been stated earlier. Hence, it can be concluded that a high pre-stress on the composite wraps enables the railgun system to endure high electromagnetic loads while maintaining structural integrity.

#### 3.3.2. Effect of Electromagnetic Load

Figure 10 provides strong evidence of the relationship between electromagnetic load and rail expansion. The latter increases as the former increases. However, the relationship is non-linear. As explained in Section 3.2, when the electromagnetic load is relatively low, the rails and insulators remain in contact, resulting in the generation of a linear curve. However, as the rails and insulators begin to separate, the stiffness of the railgun system weakens due to a decrease in the contact surface area. The extent of rail expansion increases non-linearly with the load, and the slope of the tangent in the non-linear region increases. When the rails and insulators are separated, the rails and composite barrel bear the entire electromagnetic load, generating a linear curve. However, complete separation is not permissible during launch, as it would result in the deposition of rail materials in the gap between the rails and insulators. This deposition can cause rail corrosion and ultimately lead to structural failure [35]. The electromagnetic load is usually fixed in a railgun system. Therefore, the load should serve as the foundation for the design.

#### 3.3.3. Effect of Insulator

The deformation of the insulator is primarily caused by compressive forces resulting from pre-stress on the composite barrel during the winding procedure (Section 2). It is noteworthy that external pressure is generated when pre-stress is applied to the composite wraps/barrels. The modulus and cross-sectional area of the insulators influence deformation. In a specific railgun system, the cross-sectional area can only be determined when the size of the bore can be determined. The size of the bore influences the cross-sectional area. This section focuses solely on the modulus of the insulator material.

**Figure 13:**
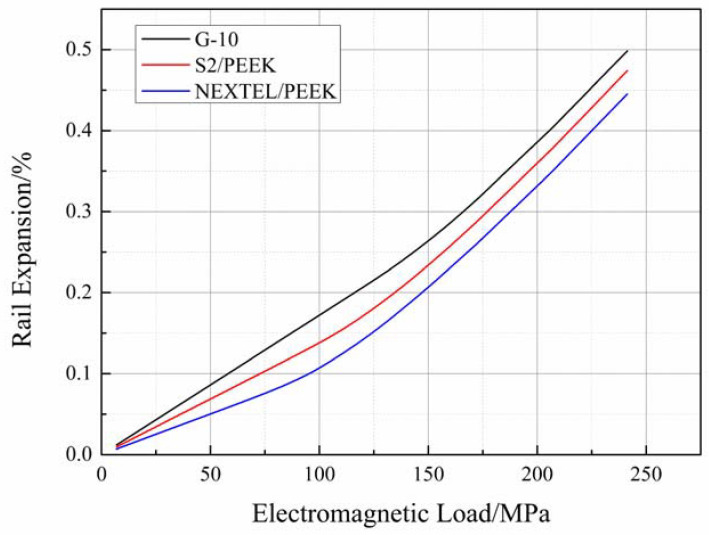
Comparison between different insulator materials.

The G-10 fiberglass blocks (24 GPa in loading direction), UD S2/PEEK laminates (52 GPa in loading direction), and UD NEXTEL/PEEK laminates (273 GPa in loading direction) were used for the investigation to satisfy the insulation requirements. The remaining components of the finite element models are identical to those described in the previous section. The simulation process was conducted under the constant tension mode with an initial pre-stress of 290 MPa. The simulation results are presented in Figure 13.

It can be observed that the curves illustrating the relationship between electromagnetic load and rail expansion for each insulator material exhibit the same characteristics as the curves discussed earlier. They consist of two linear segments and one non-linear segment. However, the transition points between the first linear–nonlinear segment and the second nonlinear–linear segment differ. The value of the load corresponding to the transition point is smaller for the structure with a stiffer insulator material. Similar observations were made for the first and the second transition point. The results are presented in Table 3. This can be explained by the fact that during the winding procedure, the extent of deformation of the insulator caused by a pre-stressed composite barrel is low when the insulator material is stiff. As the load is the same in each case and the stiffness of the rails and composite barrel is identical, the structure with stiffer insulators requires a smaller electromagnetic load to initiate the recovery of the deformation caused by pre-stress. This results in a smaller value for the transition point.

It was observed that the second set of linear segments in each curve were parallel to each other, while the first segment intersected pairwise. As mentioned in Section 2, the system remains in a fully constrained condition when the rails and insulators are in contact. Hence, the first set of linear segments intersects pairwise. Once the rails and insulators are completely separated, only the rails and composite barrels bear the electromagnetic load, and they are identical in each case. Therefore, the second set of linear segments is parallel. These observations further support the simple winding–loading model of a railgun with a pre-stressed composite barrel (Section 2).

## 4. Conclusions

A 3D finite element model was developed to analyze the factors influencing rail expansion in an EM gun. The mechanical process, which includes the winding and loading processes, was divided into two parts. Both processes were represented using simplified physical models to depict the deformation process during each phase. Special strategies such as the thermal parameter method and the element birth and death strategy were used during the use of the finite element model to capture the behavior accurately. The results obtained from the model align with previously reported experimental findings. A mechanism to explain the influence of the pre-stressed barrel on the railgun system was also proposed. Essentially, the pre-stress on the barrel plays a vital role in maintaining the overall integrity of the railgun system during loading. The finite element method was used to investigate three factors that affected rail expansion. The results indicate that the extent of rail expansion realized increases with an increase in the electromagnetic load, while it decreases as the pre-stress on the barrel decreases. Notably, a stiff insulator serves a dual purpose: it not only reduces rail expansion but also delays the separation between the rail and insulator. As a result, the railgun system can maintain its structural integrity even when subjected to a higher level of electromagnetic load. This suggests that the insulator influences the design of railgun systems. In summary, the mechanism through which pre-stress influences the railgun system is by providing initial deformation before the railgun launch, which helps to keep the railgun integral and stable throughout the launch process. 

The present work aims at the contact relationship between the rails and the insulators during the winding and loading process. Thus, we established a finite element model in which the length along Z-direction is 25.4 mm. This length matches the armature. However, the length of EM guns is generally several meters. Therefore, a finite element model for a real EM gun would be established to study the dynamic behavior of the railgun system and the contact relationship between the rails and the insulators based on the findings of the present work. The launch experiment for railguns will be also carried out in the future.

## Figures and Tables

**Table 1:** Property of rails.

Materials	Modulus/GPa	Poisson’s Ratio
Copper	104	0.34

**Table 2:** Properties of the composite used.

Item	G-10	IM7/PEEK
E_1_/GPa	24.63	172
E_2_/GPa	27.38	11.024
E_3_/GPa	11.49	11.024
μ_12_	0.194	0.3
μ_13_	0.455	0.3
μ_23_	0.518	0.3
G_12_/GPa	5.52	5.5
G_13_/GPa	12.18	5.5
G_23_/GPa	12.18	1.35

**Table 3:** Values of transition points for each researched case.

Materials of Insulator	Load Value of First Point/MPa	Load Value of Second Point/MPa
G-10	121.352	172.375
S2/PEEK	82.740	158.585
NEXTEL/PEEK	68.950	151.690

## Data Availability

Not applicable.
